# Commentary: The poverty of embodied cognition

**DOI:** 10.3389/fpsyg.2017.00845

**Published:** 2017-05-23

**Authors:** Kinga Wołoszyn, Mateusz Hohol

**Affiliations:** ^1^Institute of Psychology, Jagiellonian UniversityCracow, Poland; ^2^Institute of Philosophy and Sociology, Polish Academy of SciencesWarsaw, Poland; ^3^Copernicus Center for Interdisciplinary StudiesCracow, Poland

**Keywords:** embodied cognition, numerical cognition, research programs, methodology of cognitive science, problemshifts

According to classic cognitive science, higher cognitive processes involve amodal mental representations (Fodor, 1975), and are carried by brain regions other than sensorimotor areas (Bechtel et al., 1998). Over the last few decades, this view has been questioned. Numerous researchers argue that cognitive processes are fundamentally rooted in sensorimotor activity and that the body both constrains and enables cognition (Wilson, 2002; Clark, 2009). Such a view is called “the embodied cognition” (EC) and it is widely applied in various fields of cognitive science, from linguistics to robotics. Recently, Goldinger et al. (2016), however, questioned its applicability. They emphasized that some assumptions of EC are unacceptable, and others proffer nothing new. Primarily, they claim that EC offers no useful insight into the classic problems of experimental psychology. Although, we agree with some theses presented in the article (e.g., radical embodiment that rejects the existence of mental representation is a blind alley), it appears the authors' methodological view on EC is inadequate.

## Methodological status of embodied cognition

Goldinger et al. (2016) argue that there are many classic findings which EC cannot account for, and that it is also unable to provide any empirically testable predictions. We agree that EC cannot directly illuminate phenomena such as the Stroop interference, attentional blink effect, or scores obtained in the Sternberg memory-scanning paradigm. Indeed, this would be a good argument against EC, but only if it was a single scientific theory. In our view, however, EC should be understood as a scientific research program (Lakatos, 1976).

Research programs deliver methodological heuristics, but they are not to deliver precise predictions, or indicate experimental setups (Lakatos, 1976). The given research program is identified by its “hard core,” which is not something that can be falsified. Instead, it should inspire researchers to make specific predictions (“auxiliary hypotheses”) which can build distinct theories. These theories, in turn, should be tested, adjusted, or replaced. The theory of perceptual symbols can serve as an example (Barsalou, 1999). It has been built on the “hard core” of “weak” EC, according to which “semantic representations are at least partly constituted by sensory-motor information” (Meteyard et al., 2012, p. 792), simultaneously introducing more detailed hypotheses which can be empirically tested (Wu and Barsalou, 2009). For instance, a thesis about the contribution of the body in cognition, which can appear as trivial, was a basis for Barsalou's hypothesis that sensorimotor structures are actively and systematically involved in cognitive processing via simulation.

The fact that the “hard core” cannot be falsified, however, does not imply that it is completely irreplaceable. According to Lakatos (1976), “a research programme is successful if all this leads to a progressive problemshift, unsuccessful if it leads to a degenerating problemshift (p. 241).” The fact that the EC research program does not explain the cocktail party effect, serial recall, change blindness, and many other phenomena is not evidence for its degeneration. A growing number of findings concerning phenomena such as processing of emotional concepts (Davis et al., 2017), emotional memory (Baumeister et al., 2017), metaphoric problem solving (Keefer et al., 2013), or language comprehension (Zwaan, 2014) demonstrate that EC leads to a progressive problemshift in cognitive science.

## Embodied cognition is not so poor after all

Further examples can be found in the field of mathematical cognition which seems, at first glance, to be a bastion of amodal theories of knowledge representation (Banks and Flora, 1977). The study by Dehaene et al. (1993) showed that in the parity judgment task, left-to-right readers respond to small magnitude numbers faster with their left hand and to large magnitude numbers faster with their right hand. This effect has been dubbed “Spatial Numerical Association of Response Codes” (SNARC) and has been replicated several times in various setups and groups (see Wood et al., 2008 for meta-analysis). Reaction times in this task are said to reflect the structure of mental representation of the numerical continuum, which is “stretched” from the left (small numbers) to the right side of space (greater numbers) (Dehaene, 2011).

Fischer and Brugger (2011) presented various evidence, including neuroscientific, for Spatial Numerical Associations emerging prior to the acquisition of reading. Fischer (2012) explicitly points that “numbers are embodied concepts.” This idea is further substantiated in the study by Zago et al. (2001) who showed that recollection of simple arithmetic operations activates areas analogical to those active during the learning of finger movements sequences, or manipulation of 3D objects (see also Tschentscher et al., 2012). Andres et al. (2007) proposed a theory according to which counting is realized through simulation of finger movements.

The experiments on finger counting also add to the evidence for embodiment of mathematics (Bender and Beller, 2012). There is growing evidence that finger counting is not just a transitory step in the acquisition of mathematical competences, but it may also exert its influence on number processing (Previtali et al., 2011). In particular, Klein et al. (2011) found that the structure of finger counting affects mental arithmetic performance in educated adults. Thus, despite Goldinger et al.'s opposition (2016), mental arithmetic may be illuminated by EC. Moreover, EC is the basis for training which facilitates acquisition of basic mathematical competences (Dackermann et al., 2017). Summing up, mathematical cognition which is seemingly the domain of amodal processing, turns out to be deeply rooted in bodily experiences.

## Concluding remarks

EC, like classic cognitive science (Fodor, 1975), is a scientific research program which allows for the conceptualization and understanding of cognitive processes, but it does not provide in itself direct instructions for designing experiments, neither does it enable predictions of phenomena or give explanations of previous findings (Barsalou, 2016). The same is true for the old-fashioned cybernetics (Wiener, 1948) or such grand theoretical assumptions as representationalism or computationalism. The poverty of these research programs could be discussed in the same manner. The power of each of them lies in the fact that they lead to illuminating individual aspects of cognition, and all of them will be useful as long as they lead to a progressive problemshift. Thus, the misgiving of Goldinger et al. (2016) concerning the poverty of EC seems exaggerated.

## Author contributions

KW and MH reviewed the literature and developed the theoretical stance. KW and MH wrote the manuscript. KW and MH reviewed and accepted its final version.

## Funding

MH is supported by the research grant 2014/14/E/HS1/00803 “Cognitive Science in Search of Unity” funded by National Science Centre, Poland.

### Conflict of interest statement

The authors declare that the research was conducted in the absence of any commercial or financial relationships that could be construed as a potential conflict of interest.
